# Iron chelators target both proliferating and quiescent cancer cells

**DOI:** 10.1038/srep38343

**Published:** 2016-12-07

**Authors:** Mårten Fryknäs, Xiaonan Zhang, Ulf Bremberg, Wojciech Senkowski, Maria Hägg Olofsson, Peter Brandt, Ingmar Persson, Padraig D’Arcy, Joachim Gullbo, Peter Nygren, Leoni Kunz Schughart, Stig Linder, Rolf Larsson

**Affiliations:** 1 Department of Medical Sciences, Division of Cancer Pharmacology and Computational Medicine, Uppsala University, SE-751 85 Uppsala, Sweden; 2 Department of Medical and Health Sciences, Linköping University, SE-58183 Linköping, Sweden; 3 Cancer Center Karolinska, Department of Oncology and Pathology, Karolinska Institute, SE-171 76 Stockholm, Sweden; 4 Beactica, SE-754 50 Uppsala, Sweden; 5 Department of Medicinal Chemistry, Organic Pharmaceutical Chemistry, Uppsala University, SE-751 23 Uppsala, Sweden; 6 Department of Chemistry and Biotechnology, Swedish University of Agricultural Sciences, P.O. Box 7015, SE-756 51 Uppsala, Sweden; 7 Department of Immunology, Genetics and Pathology, Section of Oncology, Uppsala University, SE-75185, Uppsala, Sweden; 8 OncoRay-National Center for Radiation Research in Oncology, TU Dresden, D-01307 Dresden, Germany

## Abstract

Poorly vascularized areas of solid tumors contain quiescent cell populations that are resistant to cell cycle-active cancer drugs. The compound VLX600 was recently identified to target quiescent tumor cells and to inhibit mitochondrial respiration. We here performed gene expression analysis in order to characterize the cellular response to VLX600. The compound-specific signature of VLX600 revealed a striking similarity to signatures generated by compounds known to chelate iron. Validation experiments including addition of ferrous and ferric iron in excess, EXAFS measurements, and structure activity relationship analyses showed that VLX600 chelates iron and supported the hypothesis that the biological effects of this compound is due to iron chelation. Compounds that chelate iron possess anti-cancer activity, an effect largely attributed to inhibition of ribonucleotide reductase in proliferating cells. Here we show that iron chelators decrease mitochondrial energy production, an effect poorly tolerated by metabolically stressed tumor cells. These pleiotropic features make iron chelators an attractive option for the treatment of solid tumors containing heterogeneous populations of proliferating and quiescent cells.

Iron is an essential nutrient that enables a plethora of biological processes including DNA replication and mitochondrial respiration. Cancer cells display increased rate of iron uptake and usage^1^. Thus, iron may have an even more fundamental role in tumor cell hemostasis than is generally appreciated. Ferrous iron is present in a cytoplasmic pool of soluble and chelatable iron, i.e. the labile iron pool^1^. Increases in the size of the labile iron pool has been reported to lead to increased tumor cell proliferation^2^. Iron is a necessary component of haem and iron-sulfur clusters, present in enzymes involved in oxidative phosphorylation (OXPHOS) and the Krebs cycle^3^. Iron is also required for the enzymatic activity of ribonucleotide reductase (RR), catalyzing the conversion of ribonucleotides to deoxyribonucleotides^4^. Indeed, several iron chelators have been shown to possess anti-cancer activity^1^,^5^,^6^,^7^,^8^,^9^.

We recently identified the small molecule VLX600 (Fig. 1A) as a candidate drug that preferentially targets quiescent cells in colon cancer 3-D multicellular tumor spheroids (MCTS)^10^. Similar to other compounds targeting quiescent cells in MCTS^11^,^12^, VLX600 affects mitochondrial function. The anti-cancer activity of VLX600 is attributed to the limited metabolic plasticity of cancer cells in hypoxic and nutritionally compromised environments, in which cells are unable to compensate for decreased mitochondrial OXPHOS by other means of energy production. This ultimately leads to a bioenergetic catastrophe and tumor cell death^13^.

In contrast to other agents that decrease the viability of MCTS such as nitazoxanide^11^, VLX600 also inhibits the proliferation of tumor cells in 2-D monolayer culture^10^. This observation prompted us to investigate the molecular mechanism of action of VLX600. We here report that VLX600 binds iron and that this property is the underlying mechanism of the ability of VLX600 to reduce cell proliferation and to decrease mitochondrial OXPHOS. We show that also other iron chelators have the ability to affect the viability of MCTS, albeit with lower potency than VLX600. The ability of iron chelators to reduce mitochondrial energy production adds to the evidence of this class of compounds as having attractive anti-neoplastic activities.

## Results

### VLX600 is an iron chelator

**Figure 1:**
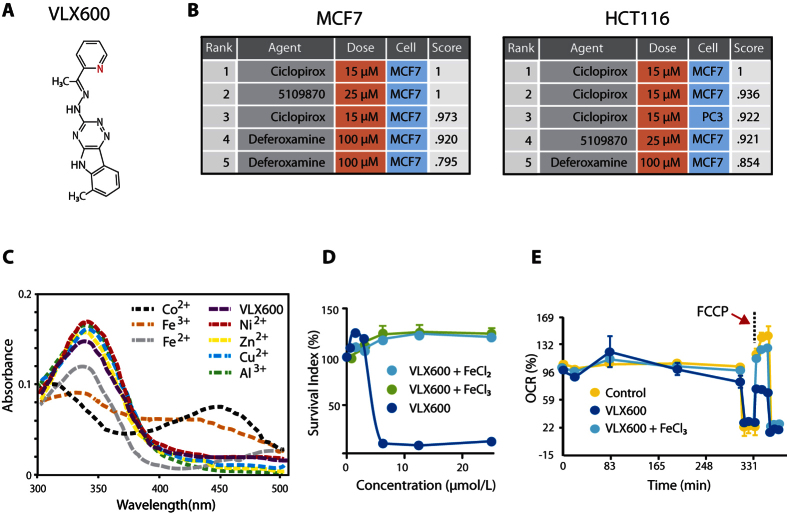
VLX600 is an iron chelator. (**A**) Molecular formula for VLX600. (**B**) Drug-specific query signatures based on the 30 most up and down regulated genes in MCF-7 cells (monolayer culture) or HCT116 cells (multicellular spheroid culture) exposed to VLX600 were uploaded to the CMAP data base to identify other compounds with similar mechanism of action. (**C**) Analysis of metal binding by VLX600 using spectrophotometry as described^16^. Note the reduction in A_340_ after addition of Fe^2+^, Fe^3+^ and Co^2+^, whereas Cu^2+^ and other metal ions do not affect A_340_. Representative of three independent experiments (**D**) Antiproliferative activity of VLX600 on HCT116 cells is abrogated by addition of iron chloride (FeCl_2_ and FeCl_3_). Cells were grown for 72 h in the presence or absence of VLX600 and iron chloride and viability was assessed by MTT assay. Mean ± S.D. (n = 4), representative repeated experiments. (**E**) The reduction of oxygen consumption by VLX600 in HCT116 cells is reversed by the addition of iron. Mean ± S.D. (n = 4), representative of two independent experiments.

The molecular structure of VLX600 is shown in Fig. 1A. The precise molecular mechanism of action of VLX600 was unknown and we therefore performed a Connectivity Map-based mechanistic exploration by examining the gene expression profile of drug-treated tumor cells^14^. We employed two different cellular models; the breast cancer cell line MCF-7 and colon carcinoma cell line HCT116, grown as 2-D monolayer and 3-D MCTS, respectively. MCF-7 cells were chosen since it is the most frequently used cell model in the Connectivity Map database. We selected MCTS HCT116 to investigate if the response is the similar when cells were grown in 3-D cell culture. The gene expression signature induced by VLX600 was most similar to that of ciclopirox olamine (CPX; 6-cyclohexyl-1-hydroxy-4-methyl-2(1H)-pyridone 2-aminoethanol), the ChemBridge compound 5109870 (2-hydroxy-3-methoxybenzaldehyde 2-pyridinylhydrazone), and deferoxamine (Fig. 1B). All of these compounds were previously described as iron chelators^15^,^16^,^17^, suggesting that the anticancer activity of VLX600 could be attributed to iron chelation and sequestering. Complex formation between VLX600 and different metals was examined using spectrophotometry (Fig. 1C). VLX600 was indeed found to form complexes with ferrous and ferric iron as well as with Co^2+^, whereas only minor changes in the UV/Vis spectra were observed with Cu^2+^, Zn^2+^, Ni^2+^ or Al^3+^ (Fig. 1C). Addition of extracellular iron (Fe^2+^ and Fe^3+^) abolished the cytotoxic activity of the compound (Fig. 1D), supporting the hypothesis of iron chelation being instrumental to biological activity of VLX600. As previously described, VLX600 is able to reduce mitochondrial OXPHOS, particularly uncoupled respiration^10^. We found that addition of iron chloride restored OXPHOS capacity in cells exposed to VLX600 (Fig. 1E).

### Characterization of iron chelation

**Figure 2:**
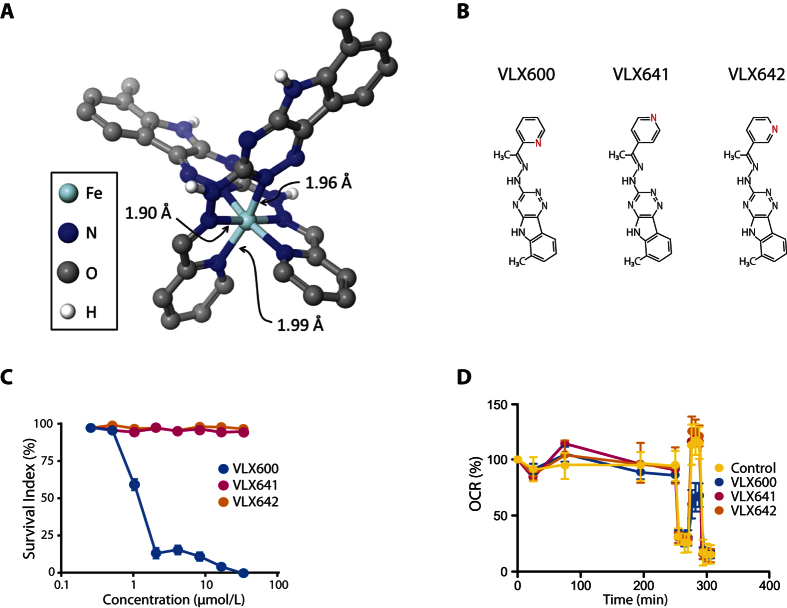
Iron binding by VLX600. (**A**) Model for binding of VLX600 to iron generated by DFT and EXAFS. (**B**) Molecular structures of VLX600, VLX641 and VLX642. The nitrogen atom in the pyridine moiety (in red) is positioned in ortho- meta- and para-position, respectively. (**C**) Cytotoxicity was completely abrogated (HCT116 colon cancer cell line, FMCA, 72 hours) when the nitrogen atom in the pyridine is placed in meta- or para position (VLX641 and VLX642). Data is based on four independent experiments, were each concentration is tested in quadruplicates (mean ± S.E.M). (**D**) The ability of VLX600 to reduce oxygen consumption is dependent on the position of the nitrogen in the pyridine ring. Mean ± S.D. (n = 4), representative of three independent experiments.

By means of density functional theory (DFT) calculations on Fe(II)(VLX600)_2_, the N2 nitrogen in the 1,2,4-triazine moiety was found to preferentially coordinate iron over the N4 nitrogen by as much as 18 kcal/mol (Fig. 2A). Using this coordination mode, the geometries were calculated for both oxidation states and spin states of Fe(VLX600)_2_ (Supplementary Table 1). Extended X-Ray Absorption Fine Structure (EXAFS) confirmed that iron(II) binds two VLX600 ligands to form a bis-complex where iron binds six nitrogen atoms in a pseudo-octahedral configuration at very short Fe-N bond distances, 1.90 and 1.915 Å for iron(II) and iron(III), respectively. This is in good agreement with reported iron(II)-terpyridine complexes, Supplementary Table 3 and the DFT calculated distances. Furthermore, Fe-C/N distances at ~2.80, 3.35 and 4.05 Å were identified and fit with distances observed in the iron(II)-terpyridine complexes and those in the calculated model presented in Fig. 2A. The very short Fe-N bond distances are consistent with both iron(II) and iron(III) being present as low-spin complexes, and with significant back-bonding for iron(II) making the bond distances even shorter and the complex being significantly stabilized. The refined structure parameters are given in Supplementary Table 2, and the model fits of the experimental EXAFS and corresponding Fourier transforms (Supplementary Figures 1 and 2, respectively). The positions of the absorption edges clearly show that the VLX600 complexes of both iron(II) and iron(III) in principle remain in their oxidation states after 48 hours of storage, see Figure S3. The iron(III) complex seems to be completely stable, while there is maybe a sign that the iron(II) complex is partly oxidized as seen in the slightly longer mean Fe-N bond distance (Supplementary Table 2), and the somewhat different shape of the absorption edge, see Figure S3. The pre-edge at 7111–7114 eV is a strong indicator of the oxidation state of iron^18^. However, in these cases the pre-peak is observed at 7112 eV for both complexes, likely due to that they are low-spin complexes and with significant back-bonding in the iron(II) VLX600 complex, Figure S3.

The chemical structure of VLX600 was modified by altering the position of the nitrogen in the pyridine moiety (VLX641 and VLX642; Fig. 2B). According to the model describing the coordination mode (Fig. 2A), this modification would result in loss of tridentate iron binding and, thus, biological activity of the molecules. Indeed, the VLX600 analogues showed no cytotoxic activity (Fig. 2C), suggesting limited off-target effects of the molecular scaffold in this biological system. Furthermore, the chemical alterations abolished the effect on mitochondrial respiration (Fig. 2D).

### Inhibition of tumor cell proliferation by VLX600

**Figure 3:**
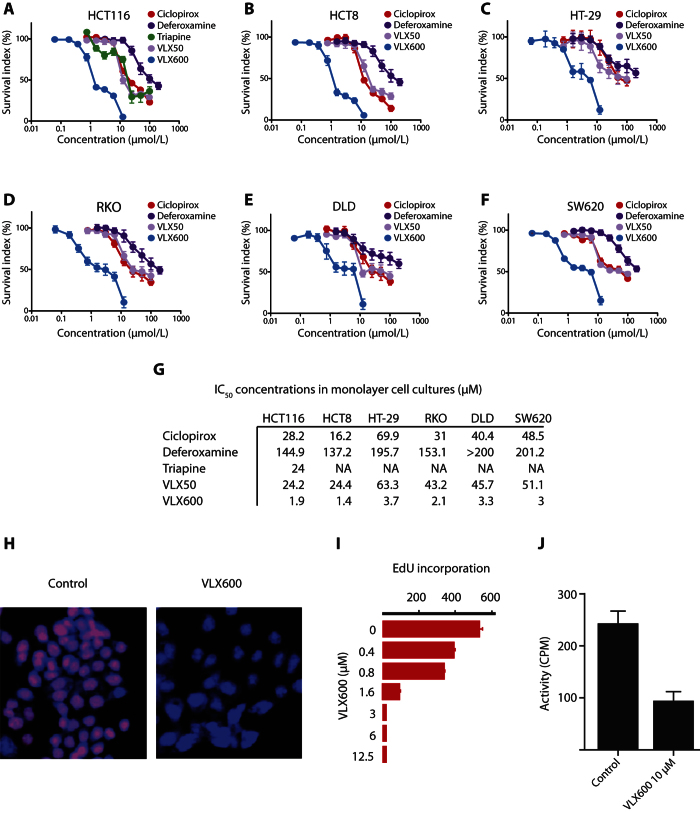
VLX600 is more potent compared to other iron chelators in reducing tumor cell proliferation. (**A–F**) Six different colon cancer cell lines (HCT116, HCT8, HT-29, RKO, DLD and SW620) were tested exposed to the indicated concentrations of VLX600 and viability was determined by FMCA at 72 hours. All concentrations tested in quadruplicate, experiments repeated three times (except HT-29, repeated twice), graphs indicate mean ± S.E.M. (**G**) Summary of IC_50_ concentrations from the data in (**A–F**). (**H**) VLX600 reduces DNA synthesis in HCT116 cells as evidenced by reduced EdU (5-ethynyl-2′-deoxyuridine) incorporation after 24-hour treatment, mean + S.D., (n = 3) representative of repeated experiment. (**C**) VLX600 inhibits ribonucleotide reductase activity *in vitro.* Results are shown as mean + S.D. (difference significant at the level of p < 0.002). Results are expressed as percentage of the untreated control and shown as mean + SD from repeated experiments where each condition was tested in duplicate wells (n = 2).

VLX600 was identified in a screen for compounds active on 3-D tumor spheroids but also shows antiproliferative activity on colon cancer cell lines in monolayer culture^10^. We examined the effect of VLX600 on a number of colon cancer cell lines and generally found IC_50_ values in the order of 1 μM (Fig. 3A–G). VLX600 was more potent than other iron chelators (i.e. Triapine (3-aminopyridine-2-carboxaldehyde-thiosemicarbazone)^8^, CPX, VLX50^19^, and deferoxamine) (Fig. 3A–G). DNA synthesis of colon cancer cells grown in monolayer culture was inhibited as evidenced by decreased incorporation of 5-ethynyl-2′-deoxyuridine (EdU) (Fig. 3H,I). The catalytic activity of ribonucleotide reductase is dependent on an iron-binding site in the M2 subunit of the enzyme and iron chelators have been found to inhibit this enzyme^20^. As expected, VLX600 inhibited ribonucleotide reductase activity *in vitro* (Fig. 3J).

### Inhibition of mitochondrial activity and spheroid viability by iron chelators

**Figure 4:**
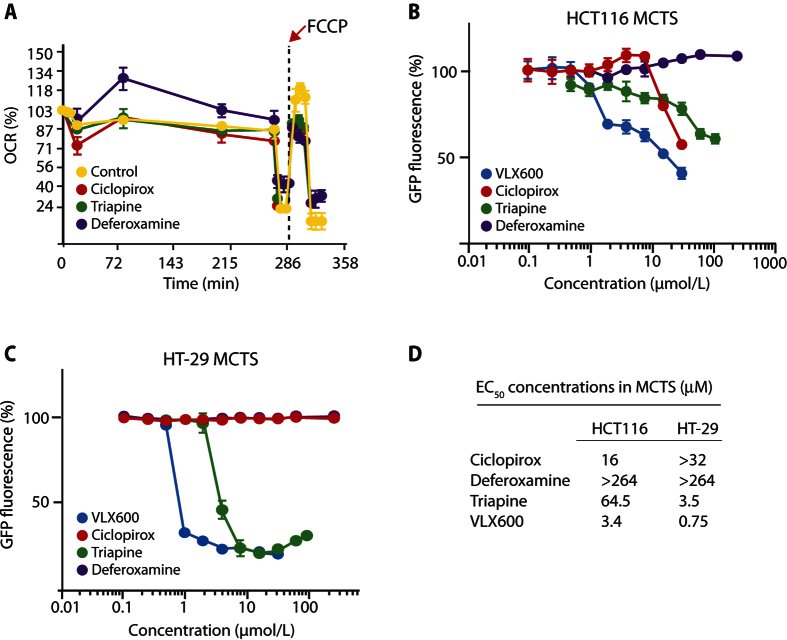
Iron chelators impact oxidative phosphorylation and cell viability. (**A**) Iron chelators diminish respiratory reserve capacity in monolayer cell cultures. Shown are Seahorse recordings of oxygen consumption of HCT116 cells exposed to the different compounds shown. Samples were run in quadruplicates, all 3 iron chelators tested induced a lower level of uncoupled respiration (p < 0.005 for Triapine and ciclopirox; p < 0.0005 for deferoxamine). Chelators were added to the culture medium at the beginning of the experiment (0 minutes). Mean ± S.D. (n = 4), representative of three independent experiments. (**B,C**) HCT116 MCTS and HT-29 MCTS were treated with different iron chelators, and GFP fluorescence was used as a surrogate marker of viability, 96-hours treatment. Mean ± S.D. (n = 6), representative of repeated experiment. (**D**) Summary of effective concentrations 50 (EC_50_) from the data in B,C.

We examined whether also other iron chelators have the ability to decrease mitochondrial oxygen consumption. Indeed, CPX, Triapine and deferoxamine all decreased uncoupled OXPHOS (Fig. 4A). CPX and Triapine reduced HCT116 MCTS viability (i.e. GFP signal; previously validated to be a reliable surrogate marker of viability^11^), although with considerably lower potency compared with VLX600 (Fig. 4B). MCTS formed from HT-29 were only affected by VLX600 and higher concentrations of Triapine whereas deferoxamine did not show detectable activity even at concentrations >300 μM. Compound lipophilicity is particularly important for activity in MCTS systems^21^ as well as in solid tumors *in vivo*^22^. The higher 3-D cytotoxic potency of VLX600 compared to other iron chelators examined here may at least be partially explained by the higher lipophilicity of VLX600 (XlogP ~ 3) (Supplementary Table 4).

### Decreased hypoxia in spheroids and in tumors

**Figure 5:**
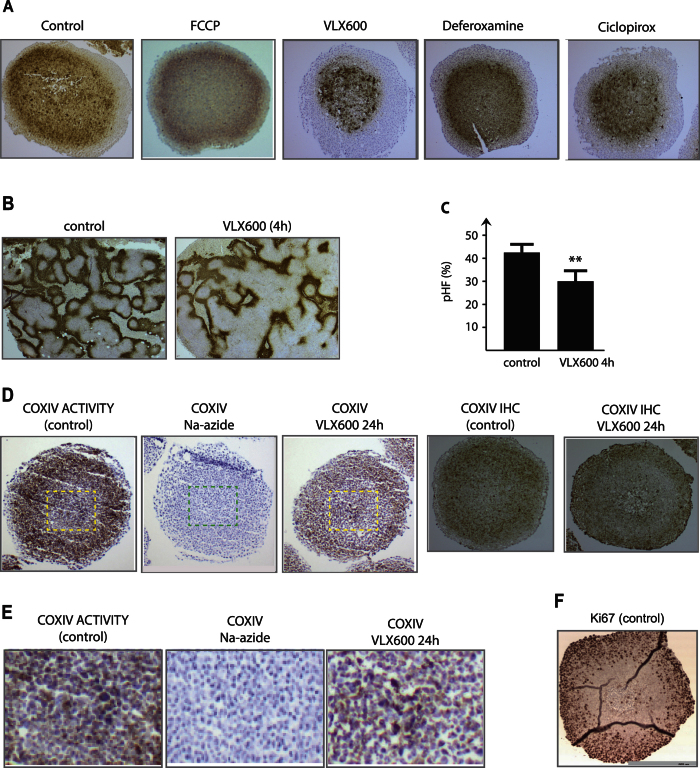
Effect of VLX600 on hypoxia and cytochrome oxidase activity. (**A**) VLX600 and ciclopirox, but not deferoxamine, reduces MCTS hypoxia. Spheroids (HCT116) were exposed to vehicle (DMSO), 6 μM VLX600, 6 μM deferoxamine or 6 μM ciclopirox. Spheroids were treated with pimonidazole for 1 h prior to fixation, sectioned and stained pimonidazole adducts to detect hypoxia. (**B**) VLX600 reduces hypoxia *in vivo*. HCT116 tumors in SCID mice were allowed to grow to a size of 200 mm^3^ before mice were injected with VLX600. Pimonidazole was injected after 3 hours and the animals sacrificed after 4 hours. Tumors were sectioned and stained for pimonidazole adducts. (**C**) Quantification of pimonidazole hypoxic fraction in the experiment. The pimonidazole hypoxic fraction (pHF) was determined in each section and shown as average + S.D. (statistics by t-test, p < 0.01). (**D**) Determination of COXIV activity in freeze fractions using histochemistry and COXIV protein levels using immunohistochemistry in control and VLX600-treated MTCS. Na-azide (2 mM) was added to the histochemistry reaction as a specificity control where indicated. (**E**) COXIV activity in MTCS core regions (the same sections shown in (**D**) are here shown at higher magnification. (**F**) Ki67 immunohistochemistry of HCT116 spheroids (included as reference to illustrate quiescence of cells in MCTS core areas).

The level of oxygen consumption in spheroids is expected to be reflected in the degree of hypoxia^10^. Exposure of spheroids to a mitochondrial uncoupler does indeed increase the pimonidazole hypoxic fraction (pHF) whereas VLX600 decreases the pHF (Fig. 5A). Similar to VLX600, ciclopirox reduced the pHF, albeit to lower degree than VLX600 (Fig. 5A), whereas deferoxamine did not cause any observable effect (Fig. 5A). These alterations of pHF are consistent with the observed degree of changes in MCTS viability (Fig. 4B).

VLX600 is well tolerated by rodents and shows anti-tumor activity in animal tumor models which are associated with decreased proliferative indices^10^. To address the question whether VLX600 affects tumor hypoxia *in vivo*, we injected VLX600 into mice bearing HCT116 colon cancer xenografts. VLX600 exposure significantly reduced the pHF 4 hours after injection of the drug (Fig. 5B,C).

### VLX600 decreases cytochrome oxidase activity in spheroids

We and others have hypothesized that energy production in tumor cells situated in the deep tumor parenchyma is vulnerable and cannot tolerate even limited decreases in OXPHOS^10^,^11^,^12^,^13^. OXPHOS is known to be functional at oxygen concentrations as low as 0.5%^23^ but it is not known whether electron transport components are expressed in core regions of spheroids. Cytochrome oxidase (complex IV) is rate-limiting in the electron transport chain^24^ and its activity was reported to decrease under hypoxic/hypoglycemic conditions^25^. We examined the distribution of cytochrome oxidase IV (COXIV) immunoreactivity and cytochrome oxidase *in situ* activity in colon cancer spheroids (Fig. 5D). Immunoreactivity and enzymatic reactivity were strongest in the area of 50–100 μm from the spheroid surface containing Ki67-positive, proliferating cells (Fig. 5D). However, COXIV activity was clearly discerned in the core regions of the spheroids (Fig. 5D,E). Exposure to VLX600 for 24 hours led to a uniform reduction of cytochrome oxidase activity in MCTS and also to a decreased immunoreactivity for COXIV (Fig. 5D).

## Discussion

The cells in the cores of MCTS have the ability to regrow after cytotoxic therapy^26^ and have in this respect similarities to cancer initiating stem cells. Recently, several studies have identified inhibition of mitochondrial OXPHOS as a promising strategy to combat quiescent cancer cells in hypoxic and nutritionally compromised environments^10^,^11^,^27^,^28^,^29^. There is a growing body of observational evidence to support this idea. Importantly, and contrary to the Warburg hypothesis, mitochondria are the main source of ATP production in cancer cells^30^ and OXPHOS is the predominant source of ATP in also hypoxic layers of MTCS^31^. Furthermore, increased OXPHOS activity is observed in human tumors *in situ* compared with adjacent normal tissue^32^. Components involved in mitochondrial biogenesis, mitochondrial translation and mitochondrial lipid biosynthesis are transcriptionally upregulated in human breast cancer epithelial cells and downregulated in adjacent stromal cells^33^. Elevated expression of the mitochondrial markers such as TIMM17A and TOMM34 is associated with poor clinical outcome and may be predictive of higher tumor grade and metastasis^34^,^35^,^36^.

Mitochondrial inhibitors have long been reported to possess anti-tumor activity^37^,^38^,^39^. Examples of such compounds are Rho123^37^, which inhibits OXPHOS^40^, and mitochondria-targeted lipophilic cations^41^. Metformin is a commonly prescribed anti-diabetic drug that increases cellular glucose uptake^42^. Metformin inhibits mitochondrial OXPHOS, likely through inhibition of complex I activity^43^,^44^ and metformin exposure appears to diminish tumor formation in diabetic patients^45^ and in mouse animal models^46^,^47^. VLX600 is a mild inhibitor of OXPHOS, primarily inhibiting uncoupled respiration (i.e total respiratory capacity)^10^. It is interesting to note that the limited effect on OXPHOS elicited by VLX600 on monolayer cultures is associated with major effects on hypoxia in spheroids. A possible explanation for this phenomenon is that total available, albeit limited, mitochondria capacity is utilized in the cells of the core area, and that mild inhibition has large consequences in oxygen utilization and energy production.

Iron chelators have complex effects on tumor cells^1^,^5^,^6^,^7^,^8^,^9^. Inhibition of ribonucleotide reductase results in inhibition of cell proliferation by depleting deoxyribonucleotide pools and we here show that iron chelators affect OXPHOS. We previously reported that VLX600 induces HIF-1α expression^10^, an observation explained by the present finding that the drug chelates iron. Prolyl hydroxylases, that negatively regulates HIF-1α protein stability, are iron-dependent enzymes^48^ and iron chelators are known to induce HIF-1α^49^ VLX600 induces a HIF-1α-dependent shift to glycolysis in exposed cells^10^, and it is possible that this leads to further depletion of glucose in tumors and a strengthened bioenergetic catastrophe. Some iron chelators form redox-active complexes with metals, notably cupper, and this has been reported to be essential for their ability to induce apoptosis^50^. Hovever, exposure to VLX600 does not result in the generation of increased levels of reactive oxygen species in cells^10^, and the antiproliferative effects of this drug is therefore most likely due to depletion of cellular labile iron pools.

The ability of iron chelators to inhibit the proliferation and decrease the viability of heterogeneous populations of tumor cells in solid tumors opens interesting possibilities for future therapies. VLX600 is presently in a phase-I clinical trial (ClinicalTrials.gov NCT02222363) for patients with refractory advanced solid tumors which will hopefully provide proof of concept for this treatment strategy.

## Experimental procedures

### Cell culture

HCT116, HT-29, SW620, HT8, DLD, RKO and MCF-7 were obtained from the American Type Culture Collection, Manassas, VA, USA). HCT116 GFP and HT-29 GFP (human epithelial colon carcinoma cell lines constitutively expressing green fluorescent protein) were purchased from Anticancer Inc. (San Diego, CA, USA). All cell lines were sub-cultivated and grown in supplemented medium as recommended by the providers. The cell banks performed authentications by short tandem repeat analysis. No further authentication was performed in our laboratory. All experiments with purchased cell lines were performed within 6 months after resuscitation. Cells were cultured in McCoy’s 5A Modified Medium (Sigma-Aldrich) +v/v 10% inactivated fetal calf serum, antibiotics (streptomycin 50 μg/mL and penicillin 60 μg/mL) and 2 mmol/L L-glutamine at 37 °C in 5% CO_2_. MCF-7 cells were maintained in Minimum Essential Medium Eagle (M5650, Sigma-Aldrich, Stockholm, Sweden), supplemented with 10% heat-inactivated FCS (Sigma-Aldrich, Stockholm, Sweden), 2 mmol/L L-glutamine, 100 μg/mL streptomycin, 100 U/mL penicillin (Sigma-Aldrich, Stockholm, Sweden) and 1 mM sodium pyruvate (P5280, Sigma-Aldrich, Stockholm, Sweden).

### Compounds

 VLX600, VLX641 and VLX642 were were acquired from OnTarget Chemistry (Uppsala, Sweden). Deferoxamine, ciclopirox and triapine were from Sigma-Aldrich (St. Louis, MO, USA). For iron chelation experiments, Cu^2+^, Co^2+^, Al^3+^, Ni^2+^, Zn^2+^ (nitrate salts) and FeCl_2_ and FeCl_3_ were obtained from Sigma Aldrich (St Louis, MO, USA).

### Connectivity Map

The Connectivity Map (CMAP) (www.broad.mit.edu/cmap) build 02 contains genome-wide expression data for 1300 compounds (6100 instances, including replicates, different doses and cell lines). We followed the original protocol using MCF-7 breast cancer cells as described by Lamb *et al*.^14^. Briefly, cells were plated in 6-well plates at a density of 0.4 × 10^6^ cells per well and were left to attach for 24 h prior to addition of drugs. We also performed CMAP-based mechanistic exploration in MCTS HCT116. MCTS HCT116 were grown as previously described^10^ and were exposed to VLX600 (10 μM) or to vehicle control (DMSO). After 6 hours treatment, the cells were washed with PBS and total RNA was prepared using RNeasy miniprep kit (Qiagen, Chatsworth, CA). Starting from two micrograms of total RNA, gene expression analysis was performed using Genome U133 Plus 2.0 Arrays according to the GeneChip Expression Analysis Technical Manual (Rev. 5, Affymetrix Inc., Santa Clara, CA). Raw data was normalized with MAS5 (Affymetrix) and gene expression ratios for drug treated vs. vehicle control cells were calculated to generate lists of regulated genes. Filter criteria were present calls for all genes in both the VLX600 treated DMSO (vehicle) exposed control cells and an expression cut-off of at least 200 arbitrary expression units. Only probes present on HG U133A were used, for CMAP compatibility. The 30 most up and down regulated genes (i.e., probes) for each compound were uploaded into CMAP and compared to the 6100 instances in the CMAP database, to retrieve a ranked compound list. Raw and normalized expression data have been deposited at Gene Expression Omnibus with accession number GSE84051 (MCF-7) and GSE53777 (MCTS HCT116).

### Metal chelation analysis

10 μM different metals (Cu^2+^, Co^2+^, Al^3+^, Ni^2+^, Zn^2+^, Fe^2+^ and Fe^3+^) were separately mixed with 10 μM VLX600. Absorbance spectra over the range 210–550 nm were obtained on Nanodrop ND-1000 spectrophotometer (Thermo Fisher Scientific, Waltham, MA USA). The maximum absorption wavelength of VLX600 is 340 nm.

### Cytotoxicity assay

The Fluorometric Microculture Cytotoxicity Assay, FMCA, described in detail previously^51^, was used for measurement of the cytotoxic effect of library compounds and the established standard drugs. The FMCA is based on measurement of fluorescence generated from hydrolysis of fluorescein diacetate (FDA) to fluorescein by cells with intact plasma membranes. Cells were seeded in the drug-prepared 384-well plates using the pipetting robot Precision 2000 (Bio-Tek Instruments Inc., Winooski, VT). In each plate, two columns without drugs served as controls and one column with medium only served as blank. Cell viability was also monitored using the MTT assay^52^ (in Fig. 1D).

### Spheroid viability assay

The spheroid-based cytotoxicity assay was performed as described previously^11^, with minor modifications. Briefly, 5,000 HCT116 GFP cells or 5,000 HT-29 GFP cells were seeded in 50 μL of McCoy’s 5A medium (10% FBS, 2 mM glutamine, 50 μg/mL streptomycin, 60 μg/mL penicillin) into each well of 384-well U-bottom Ultra Low Attachment plates (Corning, NY). After seeding, plates were centrifuged at 1,000 rpm for 5 minutes. Subsequently, spheroids were grown for 7 days without medium change in 37 °C, 5% CO_2_. Then, drugs were administered using Echo Liquid Handler 550 (Labcyte, Sunnyvale, CA). After 96 h of drug incubation, mean spheroid GFP fluorescence was measured using ArrayScan VTI Reader (Cellomics Inc., Pittsburgh, PA, USA).

### Ribonucleotide reductase assay

Ten million (1 × 10^7^) HCT116 cells grown in 150 mm plates were exposed to indicated drugs for 24 hours. The ribonucleotide assay was performed as previously described^19^.

### Immunohistochemistry

Multicellular spheroids produced by the hanging drop method in 96 well plates were fixed in 2% buffered formalin, dehydrated, embedded in paraffin and sectioned. Each sample contained 24 spheroids (spheroids from each 96 well plate were pooled into 4 groups). The sections were deparaffinized with xylene, rehydrated and microwaved and then incubated overnight with the monoclonal primary antibodies diluted in 1% (wt/vol) bovine serum albumin and visualized by standard avidin–biotin–peroxidase complex technique (Vector Laboratories, Burlingame, CA, USA). Counterstaining was performed with Mayer’s haematoxylin. Antibody MIB-1 (against the nuclear proliferation-associated antigen Ki67) was from Immunotech SA, Marseille, France and used at a dilution 1:400.

### LogP predictions

XlogP3 values were retrieved from Pub Chem https://pubchem.ncbi.nlm.nih.gov/compound/.

### Assessment of cellular DNA synthesis

The fluorescence microscope ArrayScan V HCS system (Cellomics Inc.) was used for measurement of 5-ethynyl-2′-deoxyuridine (EdU) incorporation. HCT116 cells were seeded into 96-well plates (PerkinElmer Inc., Wellesley, MA, USA), left to attach over night, before test compounds were added. Cells were treated with VLX600 for 24 h or vehicle control. Cells were stained using Click-iT EdU HCS assay (C10354, Invitrogen, Molecular Probes Inc., OR, USA) according to the manufacturer’s instructions. Processed plates were loaded in the ArrayScan and analyzed. Images were acquired for each fluorescence channel, using suitable filters with 10x objective and in each well at least 1,000 cells were analyzed. Average total intensity in the EdU channel was measured.

### Measurement of oxygen consumption rates (OCR)

Measurements of cellular oxygen consumption were performed using a Seahorse XF analyzer as recommended by the manufacturer (Agilent Technologies, Santa Clara, CA). Briefly, cells were pleated in 100 μL culture medium in XF 24-well cell plates with indicated blank control wells. Plates were first placed at room temperate for 1 hour and then moved to an incubator for another 1–5 hours to facilitate attachment of cells. Another 150 μl of culture medium was then added to each well followed by overnight incubation. Before the OCR measurements, medium was removed from each well and replaced with 500 μL Seahorse assay media (pyruvate 1 mM, glucose 25 mM) at 37 °C without CO_2_ for 1 h. OCR were measured using an XF24 Extracellular Flux Analyzer. A cell mitochondrial stress kit from Seahorse Bioscience was used for mitochondrial stress tests and experiments were performed as described previously^10^.

### Assessment of pimonidazole adducts accumulation in mouse xenografts

Once HCT116 tumors in SCID mice had grown to a size of 200 mm^3^, mice were injected with VLX600. Pimonidazole was injected 1 hour before the animals were sacrificed. Tumors were sectioned and stained for pimonidazole adducts. Quantification was performed double blind from photographs of the stained slides.

### Computational Details

All density functional calculations were done using Jaguar 8.5^53^ (Schrodinger, Inc., New York, NY, 2014). Structures were optimized using the LACVP* basis set^54^ and the M06 functional^55^. Single-point energy calculations were performed using the LACVP** basis set with a PBF solvation model^56^ for water using geometries from M06/LACVP*. C2 symmetry was used in all complexes where applicable.

### Statistics

IC_50_-values and EC_50_-values (inhibitory/effective concentration 50%) were calculated by using non-linear regression and a standard sigmoidal dose-response model in the GraphPad Prism program (GraphPad Software, Inc. San Diego, CA, USA). Statistical significance was assessed using Student’s t-test (Microsoft Excel).

## Additional Information

**How to cite this article**: Fryknäs, M. *et al*. Iron chelators target both proliferating and quiescent cancer cells. *Sci. Rep.* **6**, 38343; doi: 10.1038/srep38343 (2016).

**Publisher's note:** Springer Nature remains neutral with regard to jurisdictional claims in published maps and institutional affiliations.

## Supplementary Material

Supplementary Information

## References

[b1] Torti S. V. & Torti F. M. Iron and cancer: more ore to be mined. Nature reviews. Cancer 13, 342–355 (2013). 23594855 10.1038/nrc3495 PMC4036554

[b2] Kakhlon O., Gruenbaum Y. & Cabantchik Z. I. Ferritin expression modulates cell cycle dynamics and cell responsiveness to H-ras-induced growth via expansion of the labile iron pool. The Biochemical journal 363, 431–436 (2002). 11964143 10.1042/0264-6021:3630431 PMC1222495

[b3] Tong W. H. & Rouault T. A. Metabolic regulation of citrate and iron by aconitases: role of iron-sulfur cluster biogenesis. Biometals: an international journal on the role of metal ions in biology, biochemistry, and medicine 20, 549–564 (2007). 10.1007/s10534-006-9047-6 17205209

[b4] Elford H. L., Freese M., Passamani E. & Morris H. P. Ribonucleotide reductase and cell proliferation. I. Variations of ribonucleotide reductase activity with tumor growth rate in a series of rat hepatomas. The Journal of biological chemistry 245, 5228–5233 (1970). 4319235

[b5] Lui G. Y. . Targeting cancer by binding iron: Dissecting cellular signaling pathways. Oncotarget 6, 18748–18779 (2015). 26125440 10.18632/oncotarget.4349 PMC4662454

[b6] Lovejoy D. B. & Richardson D. R. Novel “hybrid” iron chelators derived from aroylhydrazones and thiosemicarbazones demonstrate selective antiproliferative activity against tumor cells. Blood 100, 666–676 (2002). 12091363 10.1182/blood.v100.2.666

[b7] Whitnall M., Howard J., Ponka P. & Richardson D. R. A class of iron chelators with a wide spectrum of potent antitumor activity that overcomes resistance to chemotherapeutics. Proceedings of the National Academy of Sciences of the United States of America 103, 14901–14906 (2006). 17003122 10.1073/pnas.0604979103 PMC1595448

[b8] Shao J. . A Ferrous-Triapine complex mediates formation of reactive oxygen species that inactivate human ribonucleotide reductase. Mol Cancer Ther 5, 586–592 (2006). 16546972 10.1158/1535-7163.MCT-05-0384

[b9] Yu Y., Wong J., Lovejoy D. B., Kalinowski D. S. & Richardson D. R. Chelators at the cancer coalface: desferrioxamine to Triapine and beyond. Clinical cancer research: an official journal of the American Association for Cancer Research 12, 6876–6883 (2006). 17145804 10.1158/1078-0432.CCR-06-1954

[b10] Zhang X. . Induction of mitochondrial dysfunction as a strategy for targeting tumour cells in metabolically compromised microenvironments. Nature communications 5, 3295 (2014). 10.1038/ncomms4295 PMC3929804 24548894

[b11] Senkowski W. . Three-Dimensional Cell Culture-Based Screening Identifies the Anthelmintic Drug Nitazoxanide as a Candidate for Treatment of Colorectal Cancer. Mol Cancer Ther (2015). 10.1158/1535-7163.MCT-14-0792 25911689

[b12] Wenzel C. . 3D high-content screening for the identification of compounds that target cells in dormant tumor spheroid regions. Experimental cell research 323, 131–143 (2014). 24480576 10.1016/j.yexcr.2014.01.017

[b13] Zhang X. . Targeting Mitochondrial Function to Treat Quiescent Tumor Cells in Solid Tumors. International journal of molecular sciences 16, 27313–27326 (2015). 26580606 10.3390/ijms161126020 PMC4661878

[b14] Lamb J. . The Connectivity Map: using gene-expression signatures to connect small molecules, genes, and disease. Science 313, 1929–1935 (2006). 17008526 10.1126/science.1132939

[b15] Eberhard Y. . Chelation of intracellular iron with the antifungal agent ciclopirox olamine induces cell death in leukemia and myeloma cells. Blood 114, 3064–3073 (2009). 19589922 10.1182/blood-2009-03-209965

[b16] Coombs G. S. . Modulation of Wnt/beta-catenin signaling and proliferation by a ferrous iron chelator with therapeutic efficacy in genetically engineered mouse models of cancer. Oncogene 31, 213–225 (2012). 21666721 10.1038/onc.2011.228 PMC3257471

[b17] Donfrancesco A., Deb G., De Sio L., Cozza R. & Castellano A. Role of deferoxamine in tumor therapy. Acta Haematol 95, 66–69 (1996). 8604589 10.1159/000203951

[b18] Petit P. E., Farges F., Wilke M. & Solé V. A. Determination of the iron oxidation state in Earth materials using XANES pre-edge information. J Synchrotron Radiat 8, 952–954 (2001). 11512990 10.1107/s0909049500021063

[b19] Gullbo J. . Phenotype-based drug screening in primary ovarian carcinoma cultures identifies intracellular iron depletion as a promising strategy for cancer treatment. Biochemical pharmacology 82, 139–147 (2011). 21531212 10.1016/j.bcp.2011.04.003

[b20] Thelander L. & Graslund A. Mechanism of inhibition of mammalian ribonucleotide reductase by the iron chelate of 1-formylisoquinoline thiosemicarbazone. Destruction of the tyrosine free radical of the enzyme in an oxygen-requiring reaction. The Journal of biological chemistry 258, 4063–4066 (1983). 6300073

[b21] Fayad W. . Identification of agents that induce apoptosis of multicellular tumour spheroids: enrichment for mitotic inhibitors with hydrophobic properties. Chemical biology & drug design 78, 547–557 (2011). 21726416 10.1111/j.1747-0285.2011.01170.x

[b22] Minchinton A. I. & Tannock I. F. Drug penetration in solid tumours. Nature reviews. Cancer 6, 583–592 (2006). 16862189 10.1038/nrc1893

[b23] Rumsey W. L., Schlosser C., Nuutinen E. M., Robiolio M. & Wilson D. F. Cellular energetics and the oxygen dependence of respiration in cardiac myocytes isolated from adult rat. The Journal of biological chemistry 265, 15392–15402 (1990). 2394731

[b24] Villani G. & Attardi G. *In vivo* control of respiration by cytochrome c oxidase in human cells. Free radical biology & medicine 29, 202–210 (2000). 11035248 10.1016/s0891-5849(00)00303-8

[b25] Tomitsuka E., Kita K. & Esumi H. An anticancer agent, pyrvinium pamoate inhibits the NADH-fumarate reductase system–a unique mitochondrial energy metabolism in tumour microenvironments. Journal of biochemistry 152, 171–183 (2012). 22528668 10.1093/jb/mvs041

[b26] Durand R. E. Multicell spheroids as a model for cell kinetic studies. Cell Tissue Kinet 23, 141–159 (1990). 2192799 10.1111/j.1365-2184.1990.tb01111.x

[b27] Birsoy K. . Metabolic determinants of cancer cell sensitivity to glucose limitation and biguanides. Nature 508, 108–112 (2014). 24670634 10.1038/nature13110 PMC4012432

[b28] LeBleu V. S. . PGC-1alpha mediates mitochondrial biogenesis and oxidative phosphorylation in cancer cells to promote metastasis. Nature cell biology 16, 992-1003, 1001–1015 (2014). 10.1038/ncb3039 PMC4369153 25241037

[b29] Viale A. . Oncogene ablation-resistant pancreatic cancer cells depend on mitochondrial function. Nature 514, 628–632 (2014). 25119024 10.1038/nature13611 PMC4376130

[b30] Zu X. L. & Guppy M. Cancer metabolism: facts, fantasy, and fiction. Biochemical and biophysical research communications 313, 459–465 (2004). 14697210 10.1016/j.bbrc.2003.11.136

[b31] Mandujano-Tinoco E. A., Gallardo-Perez J. C., Marin-Hernandez A., Moreno-Sanchez R. & Rodriguez-Enriquez S. Anti-mitochondrial therapy in human breast cancer multi-cellular spheroids. Biochimica et biophysica acta 1833, 541–551 (2013). 23195224 10.1016/j.bbamcr.2012.11.013

[b32] Whitaker-Menezes D. . Hyperactivation of oxidative mitochondrial metabolism in epithelial cancer cells *in situ*: visualizing the therapeutic effects of metformin in tumor tissue. Cell cycle 10, 4047–4064 (2011). 22134189 10.4161/cc.10.23.18151 PMC3272287

[b33] Sotgia F. . Mitochondria “fuel” breast cancer metabolism: fifteen markers of mitochondrial biogenesis label epithelial cancer cells, but are excluded from adjacent stromal cells. Cell cycle 11, 4390–4401 (2012). 23172368 10.4161/cc.22777 PMC3552922

[b34] Aleskandarany M. A. . TOMM34 expression in early invasive breast cancer: a biomarker associated with poor outcome. Breast cancer research and treatment 136, 419–427 (2012). 23053644 10.1007/s10549-012-2249-4

[b35] Salhab M., Patani N., Jiang W. & Mokbel K. High TIMM17A expression is associated with adverse pathological and clinical outcomes in human breast cancer. Breast cancer 19, 153–160 (2012). 20972741 10.1007/s12282-010-0228-3

[b36] Xu X. . Quantitative proteomics study of breast cancer cell lines isolated from a single patient: discovery of TIMM17A as a marker for breast cancer. Proteomics 10, 1374–1390 (2010). 20198662 10.1002/pmic.200900380

[b37] Bernal S. D., Lampidis T. J., McIsaac R. M. & Chen L. B. Anticarcinoma activity *in vivo* of rhodamine 123, a mitochondrial-specific dye. Science 222, 169–172 (1983). 6623064 10.1126/science.6623064

[b38] Lampidis T. J., Bernal S. D., Summerhayes I. C. & Chen L. B. Selective toxicity of rhodamine 123 in carcinoma cells *in vitro*. Cancer research 43, 716–720 (1983). 6848187

[b39] Momose I. . Mitochondrial inhibitors show preferential cytotoxicity to human pancreatic cancer PANC-1 cells under glucose-deprived conditions. Biochemical and biophysical research communications 392, 460–466 (2010). 20083087 10.1016/j.bbrc.2010.01.050

[b40] Abou-Khalil S., Abou-Khalil W. H., Planas L., Tapiero H. & Lampidis T. J. Interaction of rhodamine 123 with mitochondria isolated from drug-sensitive and -resistant Friend leukemia cells. Biochemical and biophysical research communications 127, 1039–1044 (1985). 3985951 10.1016/s0006-291x(85)80049-8

[b41] Cheng G. . Mitochondria-Targeted Drugs Synergize with 2-Deoxyglucose to Trigger Breast Cancer Cell Death. Cancer research (2012). 10.1158/0008-5472.CAN-11-3928 PMC3700358 22431711

[b42] Brunmair B. . Thiazolidinediones, like metformin, inhibit respiratory complex I: a common mechanism contributing to their antidiabetic actions? Diabetes 53, 1052–1059 (2004). 15047621 10.2337/diabetes.53.4.1052

[b43] Owen M. R., Doran E. & Halestrap A. P. Evidence that metformin exerts its anti-diabetic effects through inhibition of complex 1 of the mitochondrial respiratory chain. The Biochemical journal 348 **Pt 3**, 607–614 (2000). 10839993 PMC1221104

[b44] Andrzejewski S., Gravel S. P., Pollak M. & St-Pierre J. Metformin directly acts on mitochondria to alter cellular bioenergetics. Cancer & metabolism 2, 12 (2014). 25184038 10.1186/2049-3002-2-12 PMC4147388

[b45] Evans J. M., Donnelly L. A., Emslie-Smith A. M., Alessi D. R. & Morris A. D. Metformin and reduced risk of cancer in diabetic patients. Bmj 330, 1304–1305 (2005). 15849206 10.1136/bmj.38415.708634.F7 PMC558205

[b46] Hirsch H. A., Iliopoulos D., Tsichlis P. N. & Struhl K. Metformin selectively targets cancer stem cells, and acts together with chemotherapy to block tumor growth and prolong remission. Cancer research 69, 7507–7511 (2009). 19752085 10.1158/0008-5472.CAN-09-2994 PMC2756324

[b47] Rocha G. Z. . Metformin amplifies chemotherapy-induced AMPK activation and antitumoral growth. Clinical cancer research: an official journal of the American Association for Cancer Research 17, 3993–4005 (2011). 21543517 10.1158/1078-0432.CCR-10-2243

[b48] Ivan M. . Biochemical purification and pharmacological inhibition of a mammalian prolyl hydroxylase acting on hypoxia-inducible factor. Proceedings of the National Academy of Sciences of the United States of America 99, 13459–13464 (2002). 12351678 10.1073/pnas.192342099 PMC129695

[b49] Saletta F., Rahmanto Y. S., Noulsri E. & Richardson D. R. Iron chelator-mediated alterations in gene expression: identification of novel iron-regulated molecules that are molecular targets of hypoxia-inducible factor-1 alpha and p53. Molecular pharmacology 77, 443–458 (2010). 20023006 10.1124/mol.109.061028

[b50] Lovejoy D. B. . Antitumor activity of metal-chelating compound Dp44mT is mediated by formation of a redox-active copper complex that accumulates in lysosomes. Cancer research 71, 5871–5880 (2011). 21750178 10.1158/0008-5472.CAN-11-1218

[b51] Lindhagen E., Nygren P. & Larsson R. The fluorometric microculture cytotoxicity assay. Nature protocols 3, 1364–1369 (2008). 18714304 10.1038/nprot.2008.114

[b52] Alley M. C. . Feasibility of drug screening with panels of human tumor cell lines using a microculture tetrazolium assay. Cancer research 48, 589–601 (1988). 3335022

[b53] Bochevarov A. D. . Jaguar: A High-Performance Quantum Chemistry Software Program with Strengths in Life and Materials Sciences. Int J Quantum Chemistry 113, 2110–2142 (2013).

[b54] Hay P. J. & Wadt W. R. Ab initio effective core potentials for molecular calculations. Potentials for K to Au including the outermost core orbitals. J Chem Phys 82, 299–310 (1985).

[b55] Zhao Y. & Truhlar D. G. The M06 suite of density functionals for main group thermochemistry, thermochemical kinetics, noncovalent interactions, excited states, and transition elements: two new functionals and systematic testing of four M06-class functionals and 12 other functionals. Theor Chem Acc 120, 215–241 (2007).

[b56] Marten B. . New Model for Calculation of Solvation Free Energies: Correction of Self-Consistent Reaction Field Continuum Dielectric Theory for Short-Range Hydrogen-Bonding Effects. J Phys Chem 100, 11775–11788 (1996).

