# Supplementary material for: Iron chelators target both proliferating and quiescent cancer cells
Source: Sci Rep. 2016 Dec 7;6:38343. doi: 10.1038/srep38343 (PMC5141479; doi:10.1038/srep38343)
Supplement: Supplementary Information [file srep38343-s1.pdf]

Supplementary Material for:

Iron chelators target both proliferating and quiescent cancer cells

Mårten Fryknäs[1], Xiaonan Zhang[2,3], Ulf Bremberg[4], Wojciech Senkowski[1], Maria Hägg Olofsson[3], Peter Brandt[5], Ingmar Persson[6], Padraig D'Arcy[2], Joachim Gullbo[1,7], Peter Nygren[7], Leoni Kunz Schughart[8], Stig Linder[2,3,*] and Rolf Larsson[1,*]

[1] *Department of Medical Sciences, Division of Cancer Pharmacology and Computational Medicine, Uppsala University, SE-751 85 Uppsala, Sweden;*

[2] *Department of Medical and Health Sciences, Linköping University, Linköping, Sweden;*

[3] *Cancer Center Karolinska, Department of Oncology and Pathology, Karolinska Institute, SE-171 76* St*ockholm, Sweden;*

[4] *Beactica, SE-754 50 Uppsala, Sweden;*

[5] *Department of Medicinal Chemistry, Organic Pharmaceutical Chemistry*, *Uppsala University, SE-751 23 Uppsala, Sweden;*

[6] *Department of Chemistry and Biotechnology, Swedish University of Agricultural Sciences, P.O.Box 7015, SE-756 51 Uppsala, Sweden;*

[7] *Department of Immunology, Genetics and Pathology, Section of Oncology, Uppsala University, SE-75185, Uppsala, Sweden;*

[8] *OncoRay - National Center for Radiation Research in Oncology, TU Dresden, D-01307 Dresden, Germany.*

*These authors contributed equally to this work

Correspondence to Mårten Fryknäs (marten.fryknas@medsci.uu.se)

Supplementary *Table 1.* Bond distances[*] for low and high spin complexes as calculated by DFT (M06/LACVP*).[**]

	Low spin				High spin	
	Fe(II)	Fe(II) with deprotonated VLX600	Fe(III)	Fe(III) with deprotonated VLX600	Fe(II)	Fe(III)
Fe–N_T	1.962	1.925	1.912	1.896	2.142	1.983
Fe–N_H	1.897	1.880	1.903	1.895	2.206	2.133
Fe–N_{Py}	1.987	1.978	1.986	1.968	2.197	2.040

[*]N_T is the triazine nitrogen, N_H is the hydrazine nitrogen, and N_{Py} is the pyridine nitrogen.
[**]For an assessment of the performance of M06/LACVP*, see Supplementary Table 3.

Supplementary *Table 2.* Bond distances, *d*/Å, Debye-Waller factors, σ^2/Å^2, and number of distances, *N*, for the iron(II) and iron(III) VLX600 complexes in aqueous solution as determined by EXAFS at ambient room temperature; E_o is the refined threshold energy in eV, and S_o^2 is the refined amplitude reduction factor. MS FeN_6 includes the linear multiple scattering paths within the FeN_6 entity. *F* is the error-square sum as defined in the EXAFSPAK program. Underlined values were fixed during refinement.

Interaction	*N*	*d*	σ^2	E_o	S_o^2	*F*
Iron(II), freshly prepared						
Fe-N	6	1.897(1)	0.0017(1)	7122.6(2)	0.469(4)	12.3
MS FeN_6	3*6	3.80(2)	0.012(2)			
Fe···C/N	12	2.804(2)	0.0040(2)			
Fe-C/N-N/C	24	3.045(7)	0.0085(6)			
Fe···C/N	4	3.341(2)	0.0054(2)			
Fe···C/N	8	4.00(2)	0.017(2)			
Iron(II), stored 48 hours						
Fe-N	6	1.904(1)	0.0016(1)	7122.7(1)	0.468(3)	7.9
MS FeN_6	3*6	3.81(2)	0.011(2)			
Fe···C/N	12	2.832(4)	0.0048(3)			
Fe-C/N-N/C	24	3.057(6)	0.0094(6)			
Fe···C/N	4	3.376(2)	0.0058(2)			
Fe···C/N	8	4.067(7)	0.0106(10)			
Iron(III), freshly prepared						
Fe-N	6	1.915(1)	0.0013(1)	7124.4(2)	0.496(6)	12.4
MS FeN_6	3*6	3.84(2)	0.010(2)			
Fe···C/N	12	2.833(2)	0.0036(2)			
Fe-C/N-N/C	24	3.073(7)	0.0105(7)			
Fe···C/N	4	3.361(4)	0.0062(2)			
Fe···C/N	8	4.01(2)	0.0091(1)			
Iron(III), stored 48 hours						
Fe-N	6	1.915(1)	0.0013(1)	7124.8(1)	0.514(5)	10.2
MS FeN_6	3*6	3.84(2)	0.010(2)			
Fe···C/N	12	2.836(4)	0.0044(2)			
Fe-C/N-N/C	24	3.076(7)	0.0101(7)			
Fe···C/N	4	3.374(3)	0.0068(2)			
Fe···C/N	8	4.040(7)	0.011(2)			

Supplementary ***Table 3.*** *Distances from Fe to first coordination sphere nitrogen atoms of terpyridine (Å) in the low spin state showing the accuracy of the M06/LACVP* method.*

	DANMOU‡	*M06/LACVP**
Fe(II)–$N_{a,c}$	*1.984*	*1.987*
Fe(II)–N_b	*1.891*	*1.887*

‡ A.T. Baker, H.A. Goodwin Crystal Structure of Bis(2,2':6',2"-terpyridine)iron(II) Bis(perchlorate) Hydrate *Aust.J.Chem.* **38**, 207–214 (1985), .

Supplementary *Table 4.* Iron chelators.

Name	Structure	CAS#	Molecular weight	XlogP3
VLX600	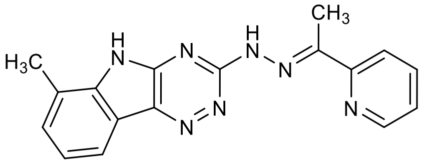	327031-55-0	317.35	2.9
Ciclopirox	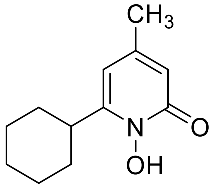	29342-05-0	207.27	2.0
VLX50, CD02750	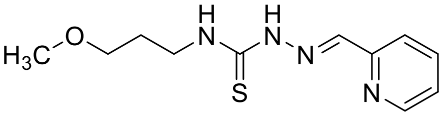	656222-45-6	252.34	1.1
Triapine	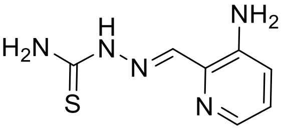	236392-56-6	195.25	0.2
Deferoxamine	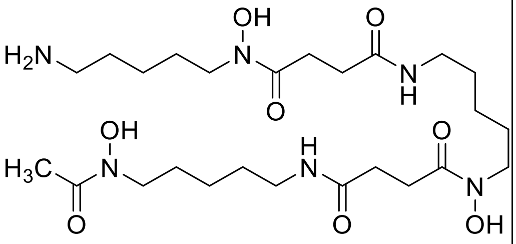	138-14-7	560.68	-2.1

Supplementary Figure 1. Fit (red) of experimental EXAFS data (black) of aqueous solutions of a/ iron(II)(VLX600)$_2$, freshly prepared (offset: 9), b/ iron(II)(VLX600)$_2$, stored in darkness for 48 hours (offset: 6), c/ iron(III)(VLX600)$_2$, freshly prepared (offset: 3), and d/ iron(II)(VLX600)$_2$, stored in darkness for 48 hours (no offset).

Supplementary Figure 2. Fit (red) of Fourier transforms from the EXAFS measurements of aqueous solutions of a/ iron(II)(VLX600)$_2$, freshly prepared (offset: 9), b/ iron(II)(VLX600)$_2$, stored in darkness for 48 hours (offset: 6), c/ iron(III)(VLX600)$_2$, freshly prepared (offset: 3), and d/ iron(II)(VLX600)$_2$, stored in darkness for 48 hours (no offset).

Supplementary Figure 3. Pre-edge peak after subtraction of smooth back-ground function of aqueous solutions of iron(II)(VLX600)$_2$, freshly prepared (black line), iron(II)(VLX600)$_2$, stored in darkness for 48 hours (green line), iron(III)(VLX600)$_2$, freshly prepared (red line), and iron(II)(VLX600)$_2$, stored in darkness for 48 hours (blue line).

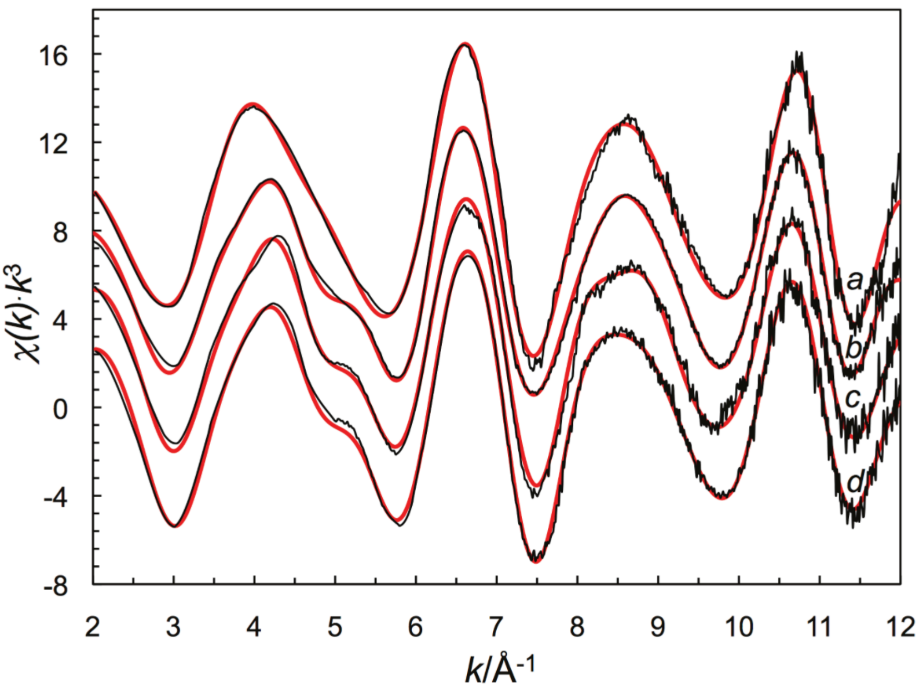

Supplementary Figure 1.

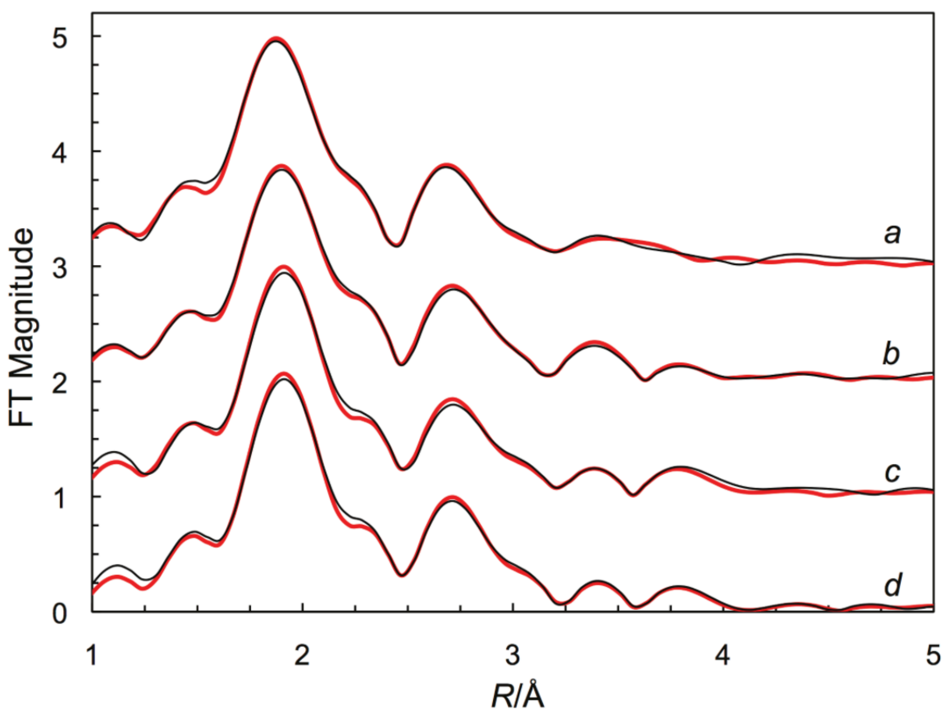

Supplementary Figure 2.

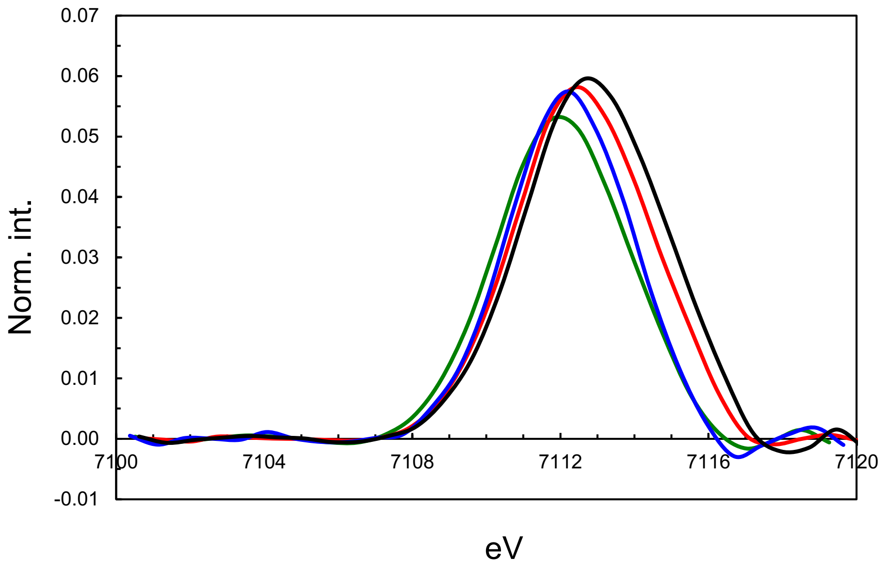

Supplementary Figure 3.
